# Nutraceutical effects of *Emblica* *officinalis* in age-related macular degeneration

**DOI:** 10.18632/aging.101820

**Published:** 2019-02-21

**Authors:** Sonali Nashine, Raj Kanodia, Anthony B. Nesburn, Girish Soman, Baruch D. Kuppermann, M. Cristina Kenney

**Affiliations:** 1 Department of Ophthalmology, Gavin Herbert Eye Institute, University of California Irvine, Irvine, CA 92697, USA; 2 Rhinoplasty Surgeon, Dr. Raj Kanodia Medical Group, Beverly Hills, CA 90210, USA; 3 Cedars-Sinai Medical Center, Los Angeles, CA 90048, USA; 4 Nisarga Biotech Pvt Ltd, Janai Malai, Satara, Maharashtra 415004, India; 5 Department of Pathology and Laboratory Medicine, University of California Irvine, Irvine, CA 92697, USA

**Keywords:** *Emblica officinalis*, *Phyllanthus emblica*, Indian gooseberry, Amla, nutraceutical, age-related macular degeneration, AMD

## Abstract

*Emblica* *officinalis* Gaetrn *(*i.e., *Phyllanthus emblica/* Indian gooseberry/ Amla) (EO) has been used extensively as a nutraceutical in several diseases since it is known to boost immunity and offers numerous health benefits such as antioxidant, anti-inflammatory, and anti-aging effects. The goal of our study was to test the hypothesis that EO will rescue human AMD RPE transmitochondrial cells from mitochondria-induced cellular damage. AMD RPE transmitochondrial cell lines were created by fusion of mitochondria DNA-deficient APRE-19 (*Rho0*) cells with platelets isolated from AMD patients, and therefore had identical nuclei but differed in mitochondrial DNA content. These AMD RPE cells were treated with EO extract followed by characterization of effects of EO using cellular and molecular assays. Herein, EO significantly improved live cell number and mitochondrial membrane potential, reduced apoptosis and oxidative stress, down-regulated *VEGF*, and up-regulated *PGC-1α*. In conclusion, EO improved cellular and mitochondrial health, thereby playing a key cytoprotective role in AMD *in vitro*. Further studies are required to examine the mechanisms that mediate the cytoprotective effects of EO.

## Introduction

*Emblica* *officinalis* Gaetrn *(Phyllanthus emblica)*, commonly known as Indian gooseberry or Amla, is an edible fruit which is borne on a deciduous tree of the same name. All parts of the *Emblica* *officinalis* (EO) tree i.e, fruits, bark, leaves, seeds, flowers, and roots are known to have medicinal properties. EO is native to the tropical and subtropical regions of Southeast Asia including India, China, Malaysia, Bangladesh, Sri Lanka, and Mascarene Island. EO is a vital medicinal plant in Ayurveda which is the ancient holistic system of medicine practiced in the Indian subcontinent.

Several health rejuvenating Ayurvedic formulations have been prepared using EO fruit (referred to as EO throughout the paper) as a primary ingredient [1]. Phytochemically, EO is composed of several bioactive compounds such as flavonoids (i.e, Quercetin, Kaempferol), phenolic compounds (i.e., gallic acid, methyl gallate, ellagic acid, trigallayl glucose), tannins (i.e., Emblicanin A and B, phyllaemblicin B, punigluconin, pedunclagin, Chebulinic acid, Corilagin, Geraniin, Ellagotannin), amino acids (i.e., glutamic acid, aspartic acid, alanine, lysine, proline, cystine), fatty acids (i.e., stearic acid, oleic acid, palmitic acid, myristic acid, linolenic acid, linoleic acid), alkaloids (i.e., Phyllantine, Phyllembein, Phyllantidine), pectin, citric acid, ascorbic acid (Vitamin C), cellulose, gum, and albumin. Based on the stage of ripening, the vitamin C content of EO varies and is the highest in ripe EO fruits (~800 mg/100 g) compared to unripe (~560 mg/100 g) or semi-ripe (~600 mg/100g) EO fruits [2].

Due to its high Vitamin C content which on an average is ~600 mg/100 g, EO is well-known as an immunity boosting food. In addition to vitamin C, EO is a rich source of antioxidants, including polyphenols, which confer EO its free radical scavenging potential [3]. A study by Carlson et al. revealed that EO has an antioxidant content of ~261.5 mmol/100 g which was substantially higher than numerous other plant-based foods and supplements that were tested using the FRAP assay in the same study [4]. Substantive evidence validates the antioxidant and cytoprotective properties of EO in several disease models including Alzheimer’s, diabetes, cardiac diseases, inflammatory disorders, hepatic diseases, atherosclerosis, cancer, and pulmonary fibrosis [5–11].

The goal of the current study was to examine and characterize the nutraceutical potential of EO in a human retinal pigment epithelial (RPE) age-related macular degeneration (AMD) transmitochondrial cybrid cell model [12]. We hypothesized that EO will rescue AMD RPE transmitochondrial cells from cellular and mitochondrial damage *in vitro*. The results of this novel study revealed the cytoprotective role of EO in AMD RPE cybrid cells in terms of of increased viability and reduction in oxidative stress and apoptosis.

## RESULTS

### EO concentration optimization

**Figure 1:**
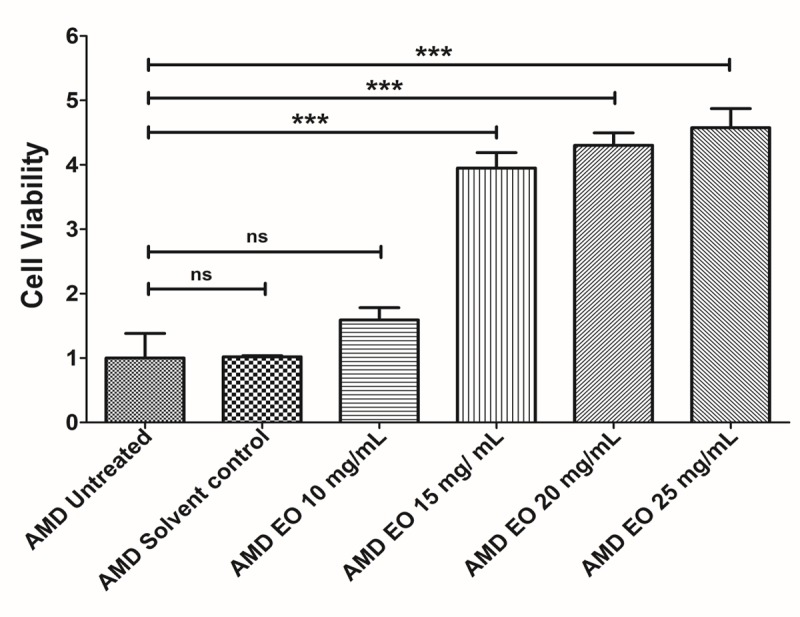
**EO concentration optimization.** Bar graph showing the effects of EO on cell death in AMD RPE cybrid cells. No difference was observed between the AMD untreated (bar 1) vs. AMD solvent control (bar 2) groups. Furthermore, no statistically significant difference was observed between untreated (bar 1) and 10 mg/mL EO-treated (bar 3) AMD cybrids. Higher viable cell numbers were observed in EO-treated AMD cybrids at concentrations of 15 mg/mL (bar 4), 20 mg/mL (bar 5), and 25 mg/mL (bar 6). *** indicates p<0.001; ns indicates non-significant p-value. Data are presented as mean ± SEM and normalized to untreated AMD cybrids which were assigned a value of 1. Experiments were performed at the 24 h time-point.

To determine the optimum working concentration of EO for all experiments, we performed an initial concentration titration experiment wherein AMD cybrids were treated with increasing doses of EO i.e, 10, 15, 20, and 25 mg/mL of EO (Figure 1) followed by measurement of viable cell numbers using MTT assay. Although compared to untreated AMD cybrids (Bar 1; 1 ± 0.382 (Mean ± SEM) arbitrary unit (a.u.), n=3), 10 mg/mL EO-treated AMD cybrids (Bar 3; 1.59 ± 0.191 a.u.; n=3) showed a 59% higher viable cell number, the difference was not statistically significant (p=0.2387). However, EO-treated cybrids showed significantly improved viable cell number at 15 mg/mL (Bar 4; 295% increase; 3.95 ± 0.240 a.u.; p<0.001; n=3), 20 mg/mL (Bar 5; 330% increase; 4.30 ± 0.193 a.u.; p<0.001; n=3), and 25 mg/mL (Bar 6; 357.5% increase; 4.575 ± 0.297 a.u.; p<0.001; n=3) compared to their untreated counterparts. No difference (p=0.96*)* in cell viability was observed between the untreated and solvent control (Bar 2; 1.018 ± 0.018 a.u.; n=3) groups. Based on these results, we chose 25 mg/mL as the optimal working concentration of EO for all experiments performed in this study.

### Effect of EO on cell viability

**Figure 2:**
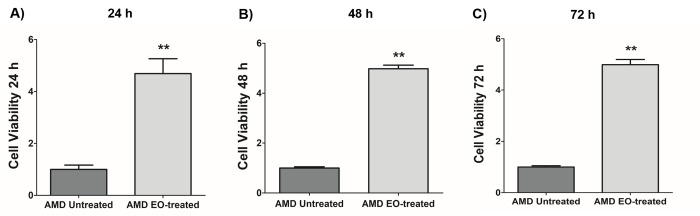
**Effect of EO on cell viability.** When treated with 25 mg/mL EO, AMD cybrid cells showed consistently increased viable cell numbers at 24 h (**A**), 48 h (**B**), and 72 h (**C**) compared to their untreated counterparts. ** indicates p<0.01. Data are presented as mean ± SEM and normalized to untreated AMD cybrids which were assigned a value of 1.

We next examined the effects of treatment of AMD RPE cybrids with 25 mg/mL EO over a time course i.e., at 24 h, 48 h, and 72 h post EO treatment (Figure 2). As anticipated, compared to their untreated counterparts, we observed significantly higher viable cell numbers in EO-treated AMD cybrids at 24 h (369% increase; AMD untreated: 1 ± 0.166 a.u., AMD EO-treated: 4.69 ± 0.571 a.u.; p=0.002; n=6) (Figure 2A), 48 h (398.1% increase; AMD untreated: 1 ± 0.049 a.u., AMD EO-treated: 4.981 ± 0.145 a.u.; p=0.008; n=5) (Figure 2B), and 72 h (398.8% increase; AMD untreated: 1 ± 0.049 a.u., AMD EO-treated: 4.988 ± 0.203 a.u.; p=0.008; n=5) (Figure 2C), suggesting that EO is able to rescue AMD cybrids from cell death [13].

### Effect of EO on Caspase-3/7 and NucLight staining

**Figure 3:**
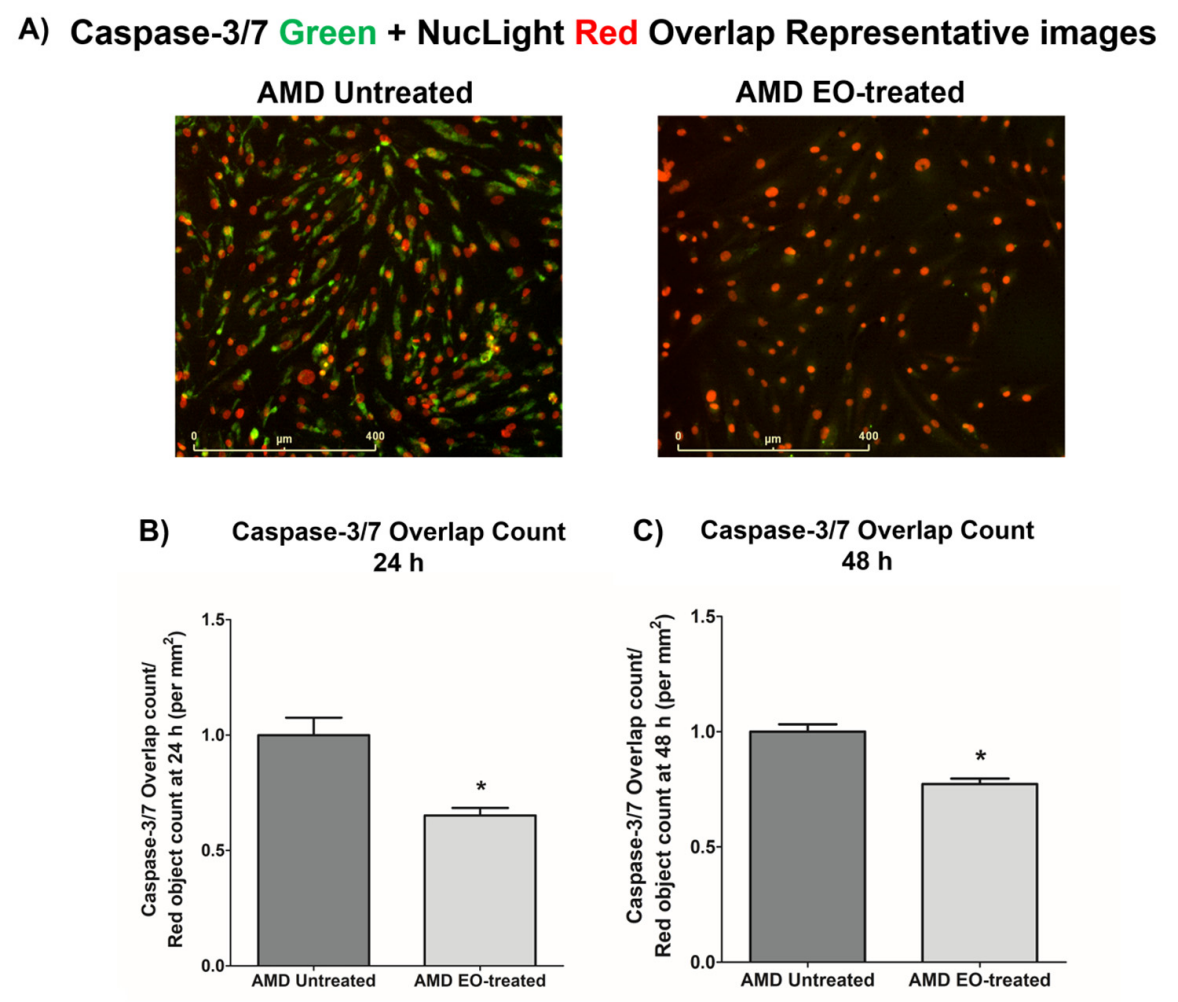
**Effect of EO on Caspase-3/7 and NucLight staining.** This figure shows representative IncuCyte live-cell images of untreated and EO-treated AMD cybrid cells stained with NucLight Red and Caspase-3/7 Green reagent (**A**) and quantitation graphs for Caspase-3/7 Green and NucLight Red staining at the 24 h (**B**) and 48 h (**C**) time points. * indicates p<0.05. Data are presented as mean ± SEM, normalized to untreated AMD cybrids, which were assigned a value of 1.

To compare the effects of EO on Caspase-3/7 activity, we morphologically monitored the kinetic activation of Caspase-3/7 using IncuCyte® live-cell imaging (Figure 3). Figure 3A shows representative overlap live-cell images of AMD RPE cybrids stained with Caspase-3/7 Green + NucLight Red reagents. The left panel represents untreated AMD cybrids and the right panel represents EO-treated AMD cybrids. We observed that EO reduced the number of Overlap object count (Yellow) (i.e., Caspase-3/7 Green + NucLight Red staining / NucLight Red object count) at 24 h (34.8% decrease; AMD untreated: 1 ± 0.076 a.u., AMD EO-treated: 0.652 ± 0.032 a.u.; p=0.029; n=4) (Figure 3B) and 48 h (22.7% decrease; AMD untreated: 1 ± 0.032 a.u., AMD EO-treated: 0.773 ± 0.023 a.u.; p=0.001, n=4) (Figure 3C). These results suggest that EO can mitigate Caspase-3/7 mediated apoptosis in AMD RPE cybrids.

### Effect of EO on *Caspase-3* and *MT-RNR2* gene expression

**Figure 4:**
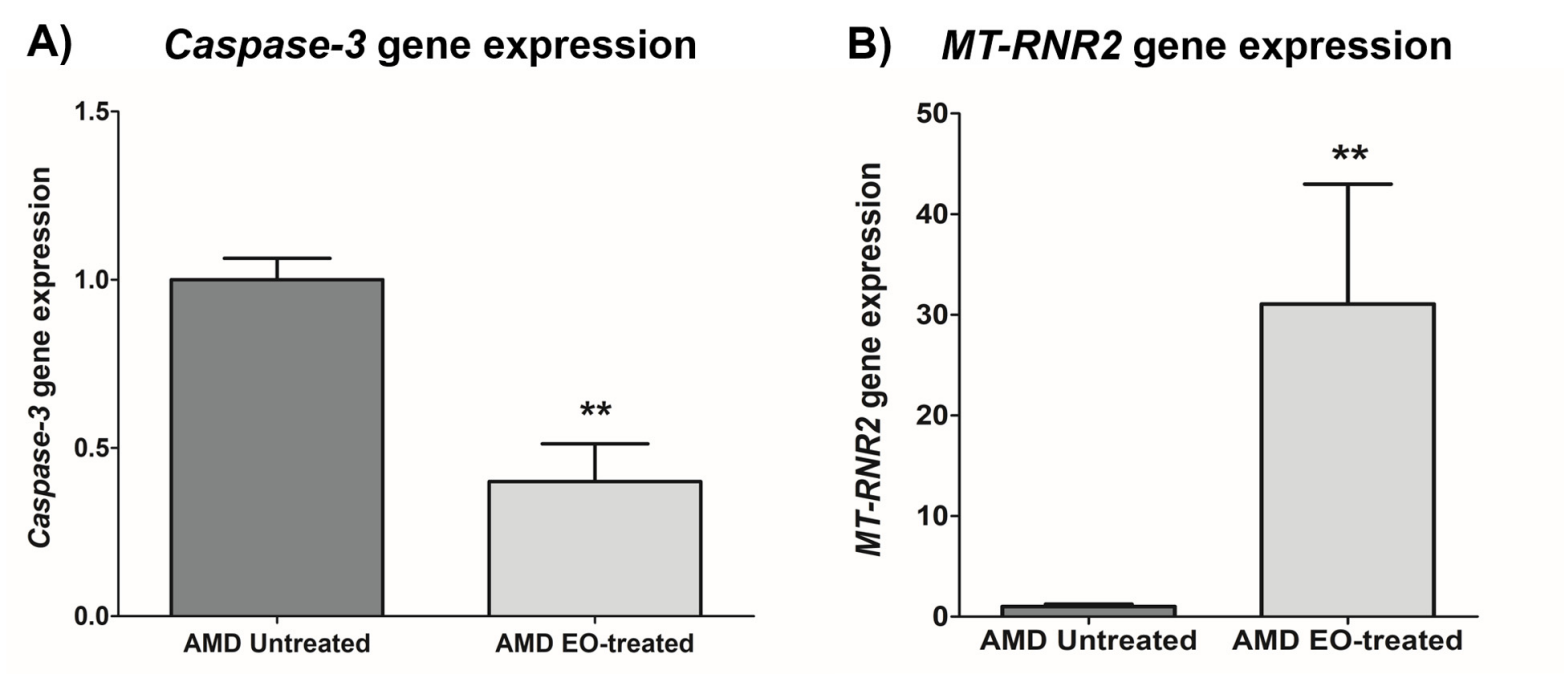
**Effect of EO on *Caspase-3* and *MT-RNR2* gene expression.** Treatment of AMD cybrids with EO reduced the gene expression of *Caspase-3* (**A**) and up-regulated *MT-RNR2* gene (**B**). ** indicates p<0.01. Data are presented as mean ± SEM and normalized to untreated AMD cybrids which were assigned a value of 1.

Treatment with EO led to significant downregulation of *Caspase-3* gene (60% decrease; AMD untreated: 1 ± 0.063 a.u.; AMD EO-treated: 0.400 ± 0.112 a.u.; p=0.008; n=5) (Figure 4A), suggesting that EO reduces caspase-3-mediated apoptosis in AMD cybrids. Moreover, upregulation of the mitochondria derived peptide (MDP)-coding *MT-RNR2* gene (3006% increase; AMD untreated: 1± 0.231 a.u.; AMD EO-treated: 31.06 ± 11.93 a.u.; p=0.008; n=5) (Figure 4B) was observed in EO-treated AMD cybrids compared to untreated cells, suggesting higher MDP production as a result of EO treatment.

### Effect of EO on ROS assay and *SOD2* gene expression

**Figure 5:**
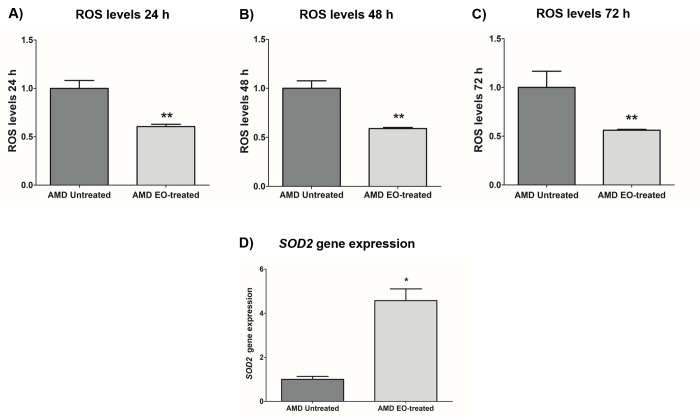
**Effect of EO on ROS assay and *SOD2* gene expression.** Addition of EO lowered ROS levels in AMD cybrids at 24 h (**A**), 48 h (**B**), and 72 h (**C**) time points. (**D**) shows increased expression of the antioxidant gene, *SOD2*, as a result of treatment with EO. ** and * indicate p<0.01 and p<0.05 respectively. Data are presented as mean ± SEM and normalized to untreated AMD cybrids which were assigned a value of 1.

To measure reactive oxygen species levels, we performed ROS assay using H2DCFDA reagent. Addition of EO to AMD cybrids reduced ROS levels at 24 h , 48 h and 72 h time points: 24 h (39.4% decrease; AMD untreated: 1 ± 0.082 a.u., AMD EO-treated: 0.606 ± 0.023 a.u.; p=0.008, n=5) (Figure 5A), 48 h (*41.1%* decrease; AMD untreated: 1 ± 0.076 a.u., AMD EO-treated: 0.589 ± 0.011 a.u.; p=0.008, n=5) (Figure 5B), and 72 h (43.9% decrease; AMD untreated: 1 ± 0.166 a.u., AMD EO-treated: 0.561 ± 0.009 a.u.; p=0.008, n=5) (Figure 5C). Furthermore, EO-treated AMD cybrids showed upregulation of *SOD2* gene compared to their untreated counterparts (357.3% increase; AMD untreated: 1 ± 0.133 a.u.; AMD EO-treated: 4.573 ± 0.533 a.u.; p=0.029; n=4) (Figure 5D). These results suggest that EO can reduce oxidative stress in AMD cybrids.

### Effect of EO on mitochondrial membrane potential and *PGC-1α* gene expression

**Figure 6:**
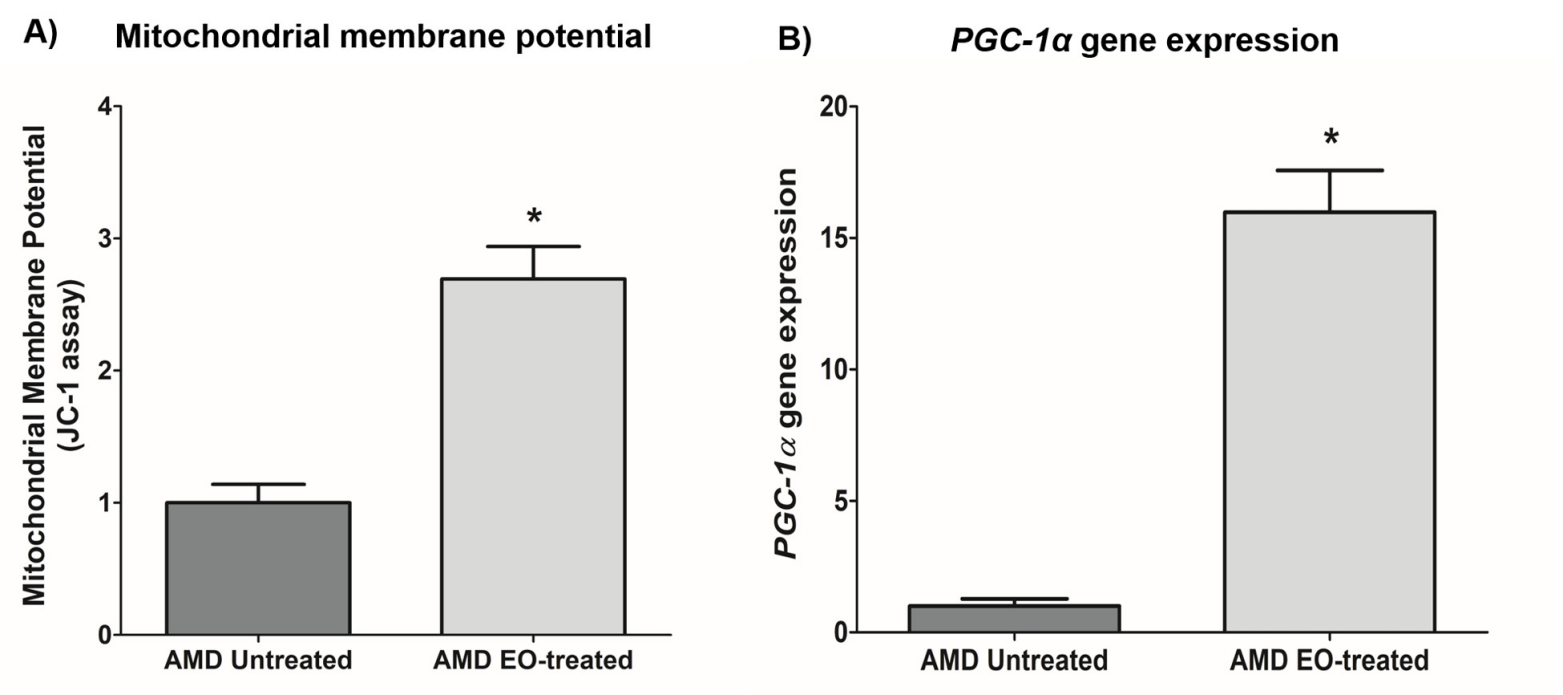
**Effect of EO on mitochondrial membrane potential and *PGC-1α* gene expression.** This figure shows increased mitochondrial membrane potential in EO-treated AMD RPE cells (**A**) and increased *PGC-1α* gene expression in EO-treated AMD cybrids (**B**). * indicates p<0.05. Data are presented as mean ± SEM and normalized to untreated AMD cybrids which were assigned a value of 1.

Compared to their untreated counterparts, EO-treated AMD cybrids showed elevated mitochondrial membrane potential at 24 h post-treatment (169.3% increase; AMD untreated: 1 ± 0.139 a.u., AMD EO-treated: 2.693 ± 0.246 a.u.; p=0.029, n=4) (Figure 6A). Moreover, EO-treated AMD cybrids showed upregulation of the *PGC-1α* gene (1498% increase; AMD untreated: 1 ± 0.277 a.u.; AMD EO-treated: 15.98 ± 1.589 a.u.; p=0.029; n=4) (Figure 6B). These results suggest that EO promotes mitochondrial health and function in AMD cybrids.

### Effect of EO on *VEGF* gene expression and on cell viability and ROS levels in amyloid-β-stressed AMD cells

**Figure 7:**
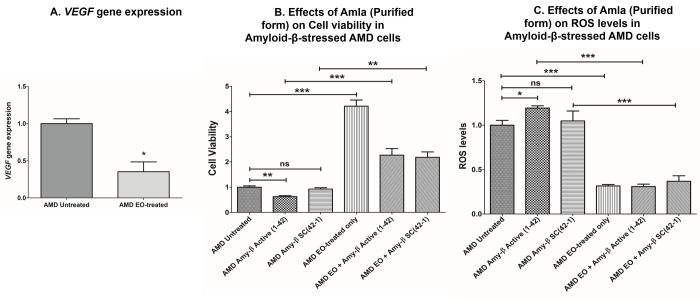
**Effect of EO on *VEGF* gene expression and on cell viability and ROS levels in amyloid-β-stressed AMD cells.** This figure showed down-regulation of *VEGF* gene in EO-treated AMD cybrids (**A**). Pretreatment with EO rescued AMD cybrids from amyloid-β-induced damage as shown by changes in cell viability (**B** (bar 2 versus bar 5) and ROS levels (**C** (bar 2 versus bar 5). *, **, and *** indicate p<0.05, p<0.01, and p<0.001 respectively; ns indicates non-significant p-value. Data are presented as mean ± SEM and normalized to untreated AMD cybrids which were assigned a value of 1.

Since VEGF has been implicated in the etiology of AMD, we next sought to compare *VEGF* gene expression between untreated and EO-treated AMD cybrids. We observed significant downregulation of *VEGF* gene in EO-treated AMD cybrids compared to untreated cybrids (64.7% decrease; AMD untreated: 1 ± 0.066 a.u.; AMD EO-treated: 0.353 ± 0.132 a.u.; p=0.029; n=4) (Figure 7A).

As amyloid-β is a component of drusen deposits formed in AMD, it is also important to determine the effect of EO treatment on amyloid-β-induced damage in AMD cybrids. Our previous studies have established that exogenously added amyloid-β_1-42_ (active form) peptides stressed AMD RPE cybrid cells [13,14]. Therefore, we analyzed the effects of EO against amyloid-β-induced damage in AMD cybrids using amyloid-β_1-42_ (active form) and amyloid-β_42-1_ (inactive scrambled control) peptides. Pretreatment with EO preserved the viable cell number and reduced ROS levels in amyloid-β_1-42_-treated AMD cybrids. In terms of cell viability (Figure 7B), there was a 263.36% increase in viable cell number in EO + amyloid-β_1-42_ treated versus amyloid-β_1-42_ alone-treated AMD cybrids (AMD amyloid-β_1-42_: 0.625 ± 0.039 a.u.; AMD EO + amyloid-β_1-42_: 2.271 ± 0.258; p<0.001; n=4) (Figure 7B – bar 2 vs. bar 5). No difference in cell viability was observed between untreated and amyloid-β_42-1_(scrambled control)-treated (0.927 ± 0.054; p=0.364; n=4) AMD cybrids. AMD cybrids treated with EO-alone (4.213 ± 0.242, n=4) served as one of the controls. Furthermore, as shown in Figure 7C, pretreatment with EO reduced ROS levels by 74.1% in EO + amyloid-β_1-42_ treated versus amyloid-β_1-42_ alone-treated AMD cells (AMD amyloid-β_1-42_: 1.193 ± 0.025 a.u.; AMD EO + amyloid-β_1-42_: 0.309 ± 0.028 a.u.; p<0.001; n=3) (Figure 7C – bar 2 vs. bar 5). No difference in ROS levels was observed between untreated and amyloid-β_42-1_(scrambled control)-treated (1.047 ± 0.112; p=0.723; n=3) AMD cybrids. EO-alone-treated AMD cybrids (0.316 ± 0.015, n=3) served as one of the controls. These results suggest that EO can rescue AMD RPE cybrid cells from cellular stress induced by amyloid-β *in vitro*.

## DISCUSSION

In the present study, we report the protective role of *Emblica officinalis* in rescuing human AMD RPE cybrid cells from damage. Herein, we analyzed the effects of exogenously added EO on the viable cell numbers, ROS levels, mitochondrial membrane potential, gene expression of *Caspase-3, SOD2, PGC-1α, MT-RNR2*, and *VEGF*, and against amyloid-β-induced toxicity in AMD RPE transmitochondrial cybrid cell lines *in vitro*.

We began our EO study by testing a varying range of concentrations of EO i.e., 10, 15, 20, and 25 mg/mL in AMD RPE cybrids *in vitro* and chose EO concentration of 25 mg/mL as the final optimal working concentration for all experiments. To our knowledge, this is the first study investigating the effects of EO on RPE cybrids containing damaged AMD mitochondria [13]. According to previous literature, varying doses of EO fruit extract are administered depending on the species, the model system, and nature of the study. For instance, Rao et al. found clinically relevant concentrations at 1–100 µg/ml of EO fruit extract to be effective in human umbilical vein endothelial cells (HUVEC) *in vitro* [15]. Yamamoto et al. demonstrated that treatment with 100-200 µg/ mL EO was effective in C2C12 myoblasts, a skeletal muscle cell line, *in vitro* [16]. However, *in vivo* and human studies required higher concentrations of EO fruit extract. For example, Lim et al. showed administration of 300 mg/kg EO to Sprague-Dawley (SD) rats was effective [17]. In diabetic studies, human subjects were given oral concentrations of EO at 1 g/mL, 2 g/mL, and 3 g/mL in water [6].

We next characterized the cytoprotective role of EO in AMD cybrids. Comparison of cell viability between untreated and EO-treated AMD cybrids demonstrated consistently higher viable cell numbers in EO-treated AMD cybrids at 24 h, 48 h and 72 h post EO-treatment. Our observations were consistent with previous studies that have highlighted the cytoprotective role of EO. For example, EO at a concentration of 500 mg/kg significantly increased cell viability, thereby rescuing splenocytes from arsenic-induced cell damage in mice [18]. Another recent finding revealed attenuation of t-BHP-induced cytotoxicity by pretreatment with EO for 48 h in a murine skeletal muscle cell line [16]. EO was shown to inhibit chromium-induced toxicity by enhancing percent cell survival and cell proliferation in an *in vitro* murine macrophage model [19]. In addition, EO prevented apoptotic cell death and enhanced cell proliferation in lymphocytes isolated from Sprague-Dawley rats [20]. Another study demonstrated the role of EO as a cytoprotectant *in vivo* [21].

It has been established previously that AMD RPE transmitochondrial cybrid cells are damaged due to diseased AMD mitochondria and undergo apoptotic cell death [13,14]. Therefore, to examine the effects of EO on apoptosis markers, we next examined the effects of EO administration on Caspase-3/7 activity using IncuCyte® live-cell imaging system and reagents. The NucLight reagent (Red) stains nuclei in live cells and the Caspase-3/7 reagent (Green) enables real-time quantification of cells undergoing caspase-3/7 mediated apoptosis. Our data revealed that EO-treated AMD cybrids had reduced Caspase-3/7 activity compared to their untreated counterparts. Moreover, AMD cybrids treated with EO had reduced expression levels of *Caspase-3* gene compared to untreated AMD cybrids. To our knowledge, this is the first study to report such EO effects in human AMD cybrids. However, addition of EO has been reported to decrease caspase-3 activity and to protect against arsenic-induced toxicity in thymocytes of mice [22]. Another study reported that co-treatment with EO reduced caspase-3 activity in splenocytes *in vitro* [18]. The same study used Annexin V/PI binding experiment to demonstrate that co-treatment with EO reduced the number of apoptotic and necrotic cells [18].

Enhanced production of reactive oxygen species (ROS) is associated with damaged mitochondria and deterioration of mitochondrial health and function [23]. AMD RPE transmitochondrial cybrid cells used in this study have higher levels of mitochondrial ROS compared to normal RPE transmitochondrial cells [13]. In the current study, we used H2DCFDA, a chemically reduced form of fluorescein, as an indicator of ROS in AMD cybrids and observed that EO induces a consistent ROS-reducing effect in AMD cybrids at 24 h, 48 h, and 72 h post treatment. This finding is critical since elevated ROS levels have been implicated in the pathogenesis of numerous aging-related diseases [24–26] including retinal diseases such as AMD, diabetic retinopathy, glaucoma, etc [27]. Quantitative RT-PCR analyses revealed that treatment with EO increased the transcript levels of *SOD2*, the mitochondrial superoxide dismutase which plays an antioxidant role in preserving cellular health in AMD [28]. This is a key finding since SOD2 deficiency contributes to oxidative damage in RPE and development of AMD pathogenesis [29]. Our current results are consistent with previous studies that have highlighted the crucial role of EO as a potential antioxidant [4,30,31] in combating oxidative stress in aging-related diseases/disorders such as diabetes, renal dysfunction, hyperlipidaemia, etc [32–34]. Elevated ROS levels can cause VEGF (Vascular Endothelial Growth Factor) activation thereby triggering angiogenesis and subsequent choroidal neovascularization in wet AMD [35,36]. In our previous studies, we found significant up-regulation of *VEGF* gene in AMD RPE transmitochondrial cybrid cells compared to age-matched normal RPE transmitochondrial cybrid cells (Data not shown). Interestingly, in the present study, EO decreased the *VEGF* gene levels in AMD cybrids, suggesting that EO might help in reducing VEGF-induced neovascularization in AMD. This data is consistent with reports by Lu et al. wherein an EO preparation inhibited VEGF-induced angiogenesis via suppression of VEGF receptor activity [37].

Using the JC-1 dye, we compared the mitochondrial membrane potential (ΔΨm) between untreated and EO-treated AMD cybrids, and found significantly higher ΔΨm in EO-treated AMD cybrids. Therefore, EO can enhance the oxidative redox state of AMD cybrids, contributing to preservation of mitochondrial integrity and function in AMD cybrids. Our data were in agreement with recent studies showing that EO positively contributes to mitochondrial health by enhancing the spare respiratory capacity [16]. QRT-PCR analyses demonstrated that EO-treated AMD cybrids had significant upregulation of *PGC-1α* (peroxisome proliferator-activated receptor gamma coactivator 1-alpha) gene compared to untreated AMD cybrids. *PGC-1α* is an important regulator of mitochondrial biogenesis and its downregulation contributes to AMD pathology [38]. Moreover, PGC-1α drives human RPE mitochondrial function and induces antioxidant capacity [39].

Drusen deposit formation under the retina is a characteristic feature in AMD [40]. Amyloid-β is a common protein found in retinal drusen deposits [41,42] and in the brains of patients with Alzheimer’s disease [43,44]. Therefore, we tested the effects of EO on amyloid-β-induced toxicity. The AMD cybrids were pretreated with EO followed by exposure to amyloid-β peptides. The EO pretreatment rescued AMD cybrids from amyloid-β-induced cellular damage as demonstrated by higher cell viability and lower ROS levels. Similar results were observed in an *in vivo* model of Alzheimer’s disease wherein oral administration of EO attenuated amyloid-induced toxicity [5].

To speculate a mechanism by which EO mediates its protective action in AMD cybrids, we measured the expression of *MT-RNR2* gene using TaqMan probes. Significant up-regulation of the MDP-coding *MT-RNR2* gene was observed in EO-treated AMD cybrids compared to the untreated cells. Since MDPs have been assigned a cytoprotective role in AMD [13,14] and other age-related diseases [45], EO-mediated cytoprotection in the AMD cybrids may be partly attributed to higher expression of the *MT-RNR2* gene. Another plausible mechanism by which EO confers its protective effects in AMD cybrids could be via aldose reductase inhibition. It is known that tannins, which are one of the components of EO, possess aldose reductase inhibitor activity [46]. Aldose reductase, an enzyme involved in glucose metabolism, has been associated with the pathogenesis of retinal diseases including diabetic retinopathy and cataract [47–50]. Chang et al. demonstrated that overexpression of aldose reductase is associated with activation of retinal microglia in mice. Since retinal microglia are immune cells that mediate inflammatory responses in the eye, their activation causes secretion of pro-inflammatory cytokines thereby contributing to the pathogenesis of eye diseases. However, suppression of aldose reductase prevents retinal microglia activation and migration, subsequently preventing ocular inflammation and disease development [51].

In conclusion, treatment with purified EO extract preserves mitochondrial and cellular health and function in human AMD RPE cybrids, implying that EO mitigates aging-related damage in AMD. Since EO extract is an over-the-counter nutraceutical and is available in both liquid and capsule forms for easy consumption, it might serve as an effective, inexpensive, and non-invasive therapeutic option for treatment of AMD. Further studies are required to fully understand the precise mechanisms that orchestrate the protective events post EO treatment in AMD cells.

## MATERIALS AND METHODS

### Human Subjects

The Institutional review board of the University of California Irvine approved research with human subjects (Approval #2003–3131). All participating subjects provided informed consent and clinical investigations were performed according to the tenets of Declaration of Helsinki.

### Cell culture

Human AMD RPE transmitochondrial cells were created by fusing mitochondria DNA-deficient APRE-19 (*Rho0*) cells with platelets isolated from AMD patient’s blood as described previously [13]. Passage 5 cells were used for all experiments (n=3-6).

### Treatment with *Emblica Officinalis* (EO)

Purified EO extract was obtained and used at a concentration of 25 mg/mL for all experiments. DMSO was used as an initial solvent. EO was subsequently dissolved in culture media for cell treatment.

### Cell viability assay (MTT assay)

The numbers of viable cells were measured using the MTT (3-(4,5-dimethylthiazol-2-yl)-2,5-diphenyltetrazolium bromide) assay (Cat.# 30006, Biotium, Fremont, CA). Cells were plated in 96-well tissue culture plates and treated with 25 mg/mL EO for 24 h and 48 h. Cells were incubated with MTT reagent at 37 °C for 1 h, followed by addition of DMSO. Signal absorbance was measured at 570 nm and background absorbance was measured at 630 nm. Normalized absorbance values were obtained by subtracting background absorbance from signal absorbance. The colorimetric signal obtained was proportional to the cell number.

### IncuCyte Live-cell imaging

The IncuCyte NucLight Rapid Red Reagent (Cat.# 4717, IncuCyte, Ann Arbor, MI) is a cell permeable DNA stain that specifically stains nuclei in live cells and enables real-time quantification of cell proliferation.

The IncuCyte Caspase-3/7 Green Apoptosis Reagent (Cat.# 4440, IncuCyte) couples the activated Caspase-3/7 recognition motif (DEVD) to a DNA intercalating dye and enables real-time quantification of cells undergoing caspase-3/7 mediated apoptosis. This reagent is an inert, non-fluorescent substrate which when added to culture medium, crosses the cell membrane where it is cleaved by activated caspase-3/7 resulting in the release of the DNA dye and fluorescent staining of the nuclear DNA.

Cells were seeded in 96-well plates at a density of 5000 – 10,000 cells/well, treated with EO, followed by staining with IncuCyte® NucLight Rapid Red (1:500) and Caspase-3/7 Green (1:1000) labeling reagents. Stained cell plates were placed into the IncuCyte® live-cell analysis system and allowed to warm to 37 °C for 30 min prior to scanning. Phase Contrast, Green, and Red channels were selected, 5 images were taken per well with an average scan interval of ~2 h until the experiment was complete. Fluorescent objects were quantified using the IncuCyte® integrated analysis software that minimizes background fluorescence.

### Reactive Oxygen Species (ROS) assay

To quantitate ROS levels, the cell-permeant H2DCFDA (2', 7’-dichlorodihydrofluorescein diacetate) was used as an indicator for ROS in cells. Stock solution of 5mM H2DCFDA was prepared in DMSO. Stock solution was then diluted in DPBS to obtain a working concentration of 10 µM. Cells were plated in 96-well tissue culture plates followed by treatment with 25 mg/mL EO. 10 µM H2DCFDA solution was added to cells and incubated for 30 min at 37 °C. H2DCFDA was then replaced with DPBS. Fluorescence which was measured at excitation 4924 nm and emission 520 nm was proportional to ROS levels in cells.

### Mitochondrial membrane potential (JC-1) assay

The JC-1 assay (Cat.# 30001-T, Biotium) uses a unique cationic dye i.e., 5,5’,6,6’-tetrachloro-1,1’,3,3’- tetraethylbenzimidazolylcarbocyanine iodide, to detect loss of mitochondrial membrane potential. JC-1 1X reagent was prepared by diluting 100X JC-1 reagent in assay buffer to 1:100 dilutions. Cells were plated in 24-well tissue culture plates followed by treatment with 25 mg/mL EO. 1X JC-1 reagent was added to cells and incubated for 15 min at 37 °C. JC-1 reagent in the wells was then replaced with DPBS and fluorescence was measured as follows: Red fluorescence (Live cells): Excitation 550 nm and Emission 600 nm; Green fluorescence (Apoptotic cells): Excitation 485 nm and Emission 535 nm. Ratio of Red/Green was used for analysis. Lower ratio corresponded to higher apoptotic/dead cell number.

### Quantitative Real-Time PCR

RNA extraction, cDNA synthesis, and qRT-PCR analysis from EO-treated AMD cybrids were performed as described previously [14]. QuantiTect Primer Assays were used to study the expression of *Caspase-3* gene (Cat. # QT00023947, Qiagen, Germantown, MD), and *SOD2* gene (Cat. # QT01008693, Qiagen). KiCqStart® SYBR® green primers were used to examine the expression of *PGC-1α* and *VEGF* genes (Cat. # kspq12012, Sigma, St. Louis, MO). Specific housekeeper genes used were *HPRT1* (Cat. # QT00059066, Qiagen) and *HMBS* (Cat. # QT00014462). TaqMan gene expression master mix (Cat. # 4369016, Life Technologies) and TaqMan gene expression assays were used to examine the expression of the *MT-RNR2* gene (Assay ID: Hs02596860_s1, Life Technologies), for which *GAPDH* (Assay ID: Hs02786624_g1, Life Technologies) was used as a housekeeper gene. Data analysis was performed using ∆∆Ct method which was calculated by subtracting ∆Ct of the AMD group from ∆Ct of the normal group. ∆Ct was the difference between the Cts (threshold cycles) of the target gene and Cts of the housekeeper gene (reference gene). Fold change was calculated using the following formula: Fold change = 2^ΔΔCt^.

### Statistical analysis

Non-parametric Mann-Whitney test (for 2 groups) or one-way ANOVA (for 3 or more groups) followed by post-hoc Tukey–Kramer test (GraphPad Prism 5.0; GraphPad Software, CA, USA) were performed to analyze data between groups. P values < 0.05 were considered statistically significant.
